# Fibro-Osseous Pseudotumor Presenting as Calcified Subcutaneous Mass Over the Achilles Tendon in a Five-Year-Old Girl

**DOI:** 10.7759/cureus.84949

**Published:** 2025-05-28

**Authors:** Nikolaos Laliotis, Panagiotis Konstantinidis, Chrysanthos Chrysanthou, Lamprini Giannakopoulou, Anestis Moumtzouoglou, Themistoklis Konstantinidis

**Affiliations:** 1 Orthopaedics, Interbalkan Medical Center, Thessaloniki, GRC; 2 Orthopaedics and Traumatology, Interbalkan Medical Center, Thessaloniki, GRC; 3 Anatomy and Surgical Anatomy, Aristotle University of Thessaloniki, Thessaloniki, GRC; 4 Radiology, Interbalkan Medical Center, Thessaloniki, GRC; 5 Pathology, Interbalkan Medical Center, Thessaloniki, GRC

**Keywords:** achilles tendon thickness, benign calcified tumor, benign tumor children, fibro-osseous pseudotumor, subcutaneous calcification

## Abstract

A calcified mass located in the subcutaneous area above the Achilles tendon of a child poses difficulty for a correct diagnosis. Differential diagnosis includes tumoral calcinosis, calcified fibroma, calcified tendinitis, and myositis ossificans. A fibro-osseous pseudotumor is a rare benign lesion that usually affects the digits. Histologically, it consists of fibrous tissue with areas of osteoid formation.

We report a child with a hard subcutaneous mass above the Achilles tendon. Radiographic assessment, including X-ray, MRI, and CT scan, revealed a calcified tumor in the posterior part of the Achilles tendon, in proximity to the sural nerve and vessel. After surgical removal of the mass, histopathology revealed fibrous connective tissue with areas of islands and trabeculae of osteoid, confirming the diagnosis of a fibro-osseous pseudotumor. A subcutaneous calcified mass in a child requires appropriate clinical and pathological investigation to ensure an accurate diagnosis.

## Introduction

A fibrous osseous pseudotumor is a rare benign lesion that typically affects the digits and presents as a nodule in the subcutaneous tissue. It is characterized by fibroblastic proliferation and osteoid formation, predominantly described in young women [1-3]. Subcutaneous calcification in children presents a challenging diagnostic problem. Tumoral calcinosis, calcified aponeurotic fibroma, calcified tendinitis, and myositis ossificans are among the rare causes of calcified soft tissue in this population [4-6]. Fibrodysplasia ossificans progressiva (FOP) represents the most severe pattern of extraskeletal osseous formation in the body [7,8].

We report a case of a five-year-old girl who presented with a hard palpable mass in the posterior part of the ankle joint, above the insertion of the Achilles tendon. We conducted X-ray evaluation, MRI, and CT scan examinations. Furthermore, we evaluated the calcium and phosphorus levels to exclude phosphate dysregulation. Surgical intervention was performed to excise the lesion, and pathological examination revealed fibrous connective tissue with fibroblastic activity and the formation of islands and trabeculae of osteoid, with a few areas of mature bone. The diagnosis of a fibro-osseous pseudotumor was confirmed, and the girl had an uneventful recovery.

## Case presentation

A five-year-old girl was referred to our pediatric orthopedic department with a history of a hard mass, first noticed by her and her family three months earlier. The child was in good general health but complained that her shoe rubbed uncomfortably against the mass.

Clinical examination revealed a firm, well-defined but irregularly contoured mass located on the posterior aspect of the distal left Achilles tendon. The overlying skin appeared normal in color and texture. On palpation, the mass felt similar to a shard of glass embedded in the subcutaneous tissue.

**Figure 1:**
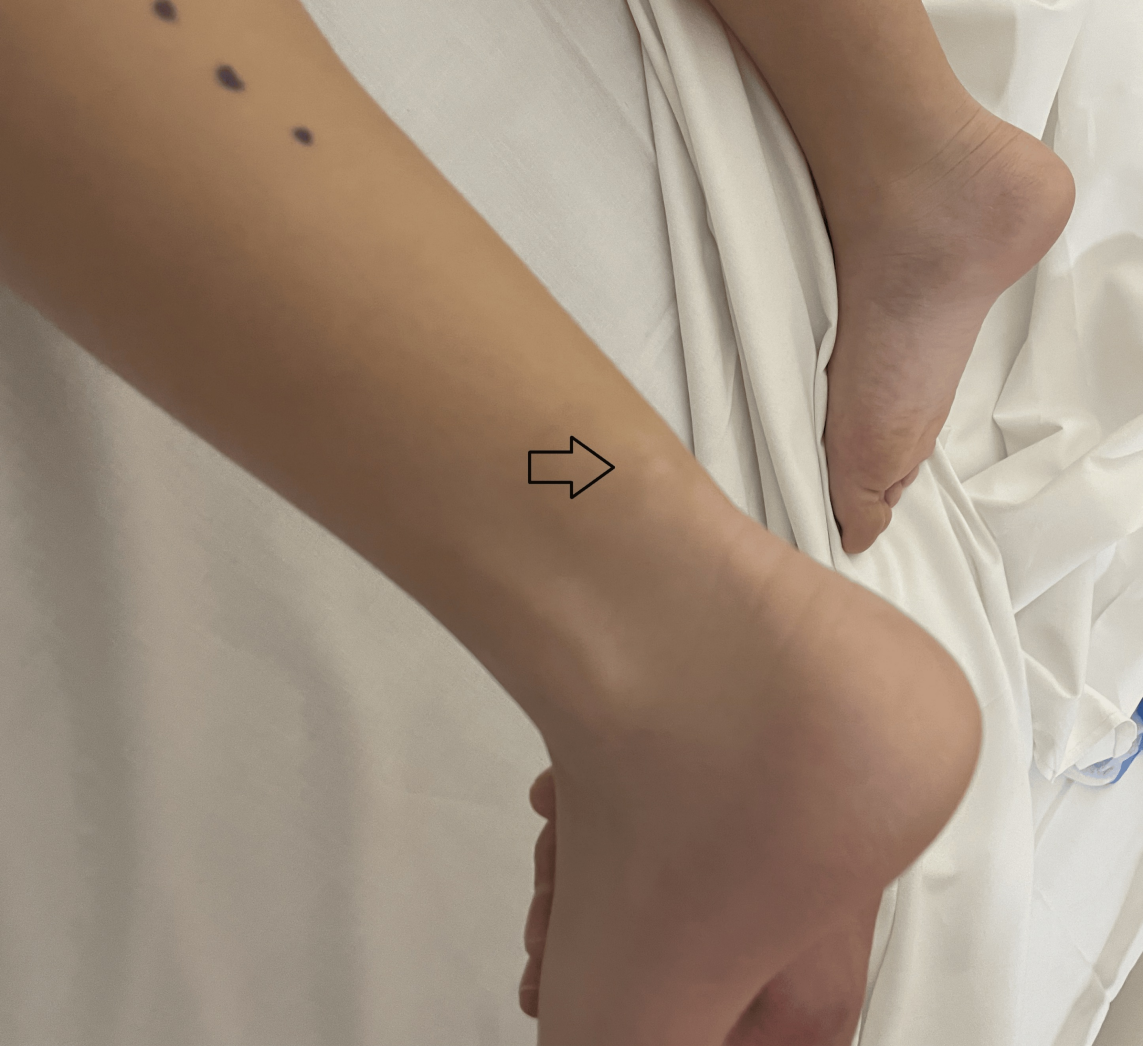
Clinical picture with the arrow pointing to the subcutaneous swelling.

The patient demonstrated full ankle range of motion and preserved foot sensation. However, she reported discomfort in the affected leg when asked to walk on tiptoes. The shape and alignment of her toes were normal (Figure 1).

**Figure 2:**
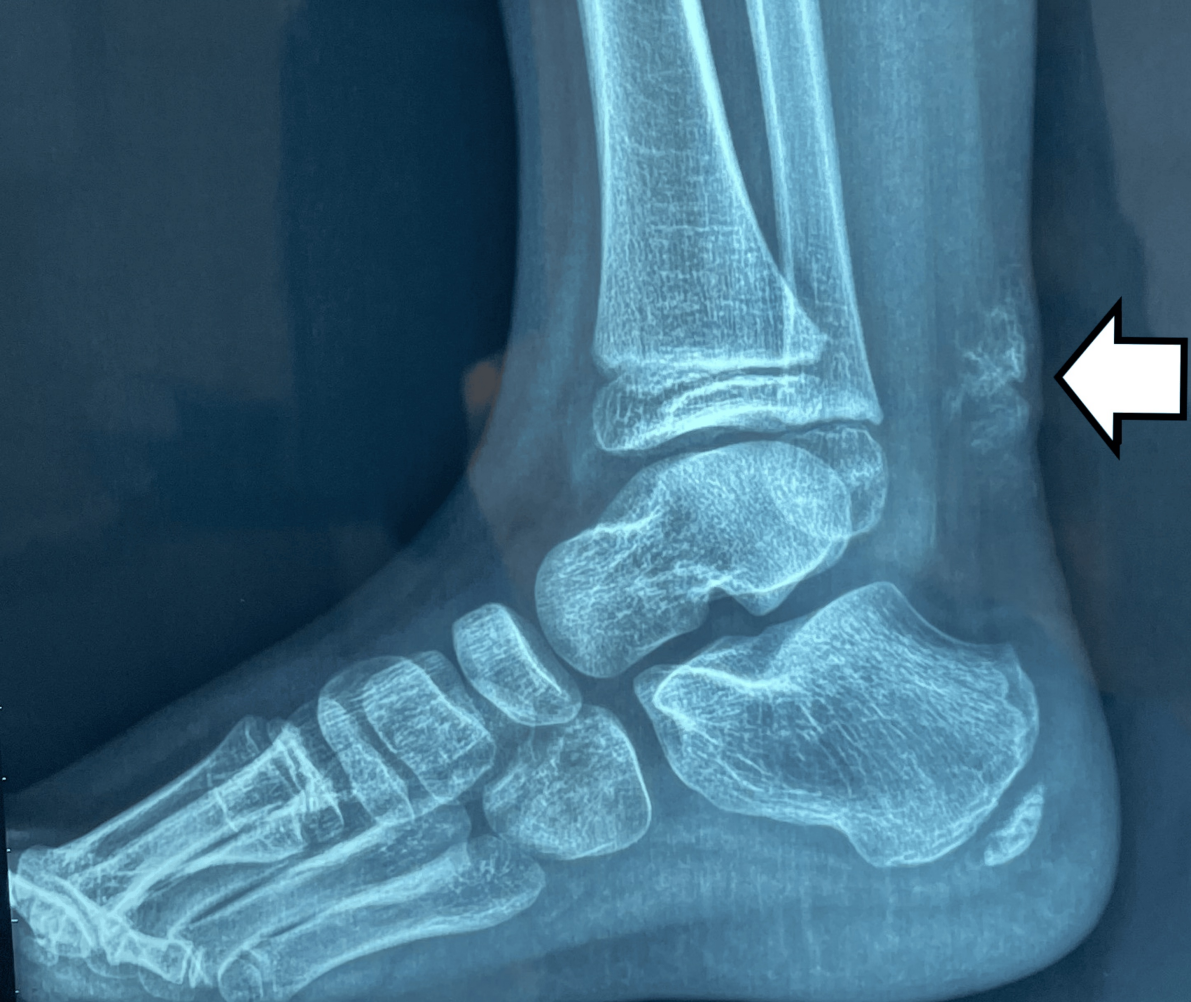
X-ray showing irregular calcification in the subcutaneous tissue, overlying the Achilles tendon.

Radiological examination revealed diffuse irregular calcification within the subcutaneous tissue overlying the Achilles tendon, with normal alignment of the ankle and foot bones (Figure 2).

**Figure 3:**
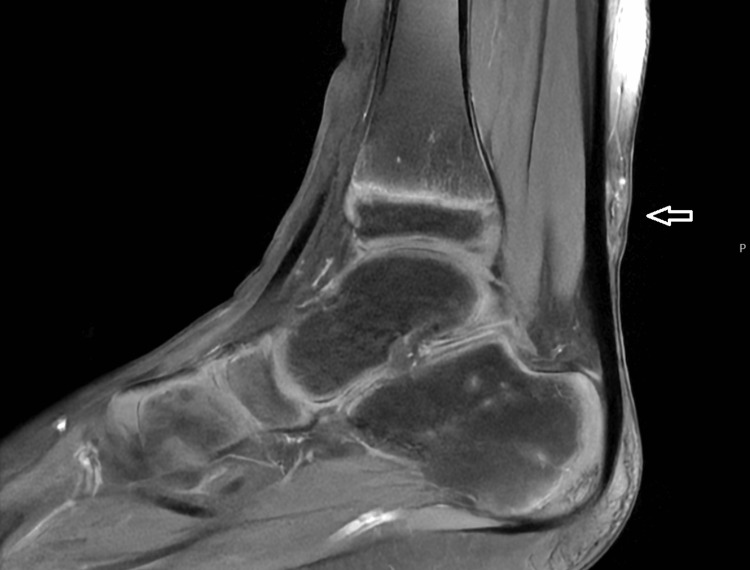
MRI scan on the sagittal plane showing a soft tissue subcutaneous mass overlying the lower third of the Achilles tendon, with areas of lower signal intensity on T1. The image demonstrates calcified tissue with mild enhancement after contrast medium administration

**Figure 4:**
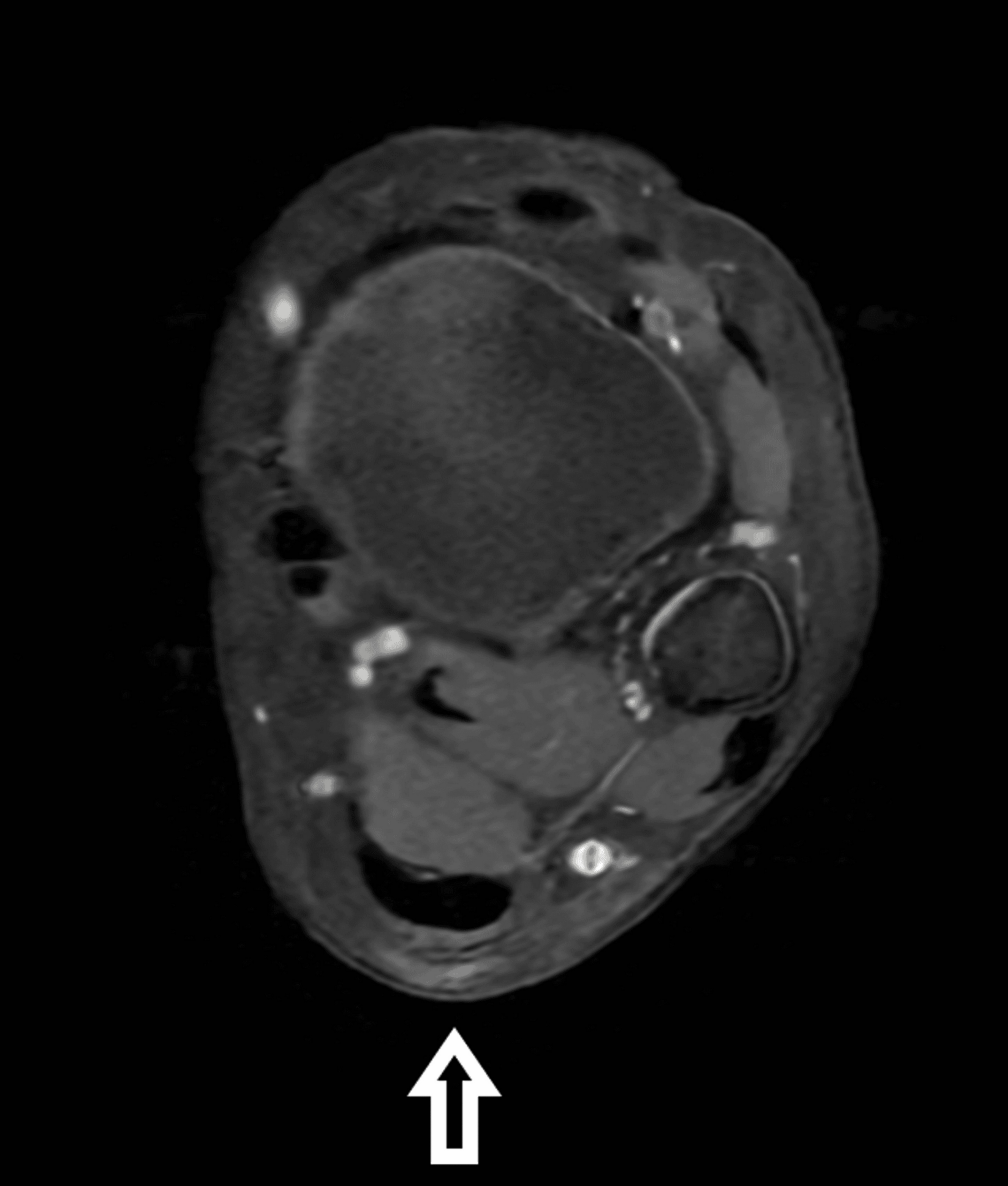
MRI scan on the transverse plane showing a soft tissue subcutaneous mass overlying the lower third of the Achilles tendon, with areas of lower signal intensity on T1.

MRI examination confirmed the presence of a lesion overlying the Achilles tendon, adjacent to the sural nerve and vein. The lesion had lower signal intensity in both T1 and T2 images, with mild enhancement after contrast administration. The lesion had an irregular shape and measured 1.6 cm in size (Figures 3, 4).

**Figure 5:**
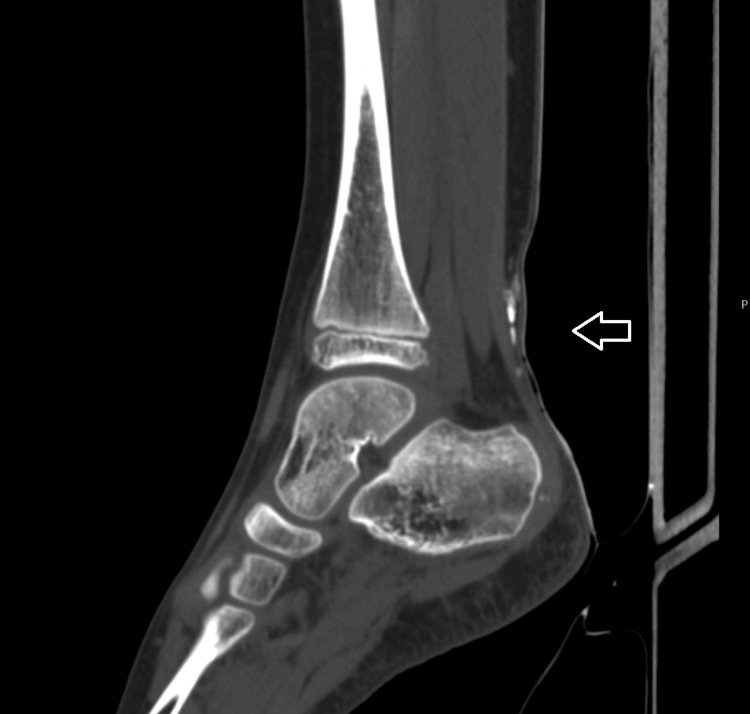
CT image on the sagittal plane showing a thin calcified lesion, located above the Achilles tendon, extending toward the sural nerve and vessel.

**Figure 6:**
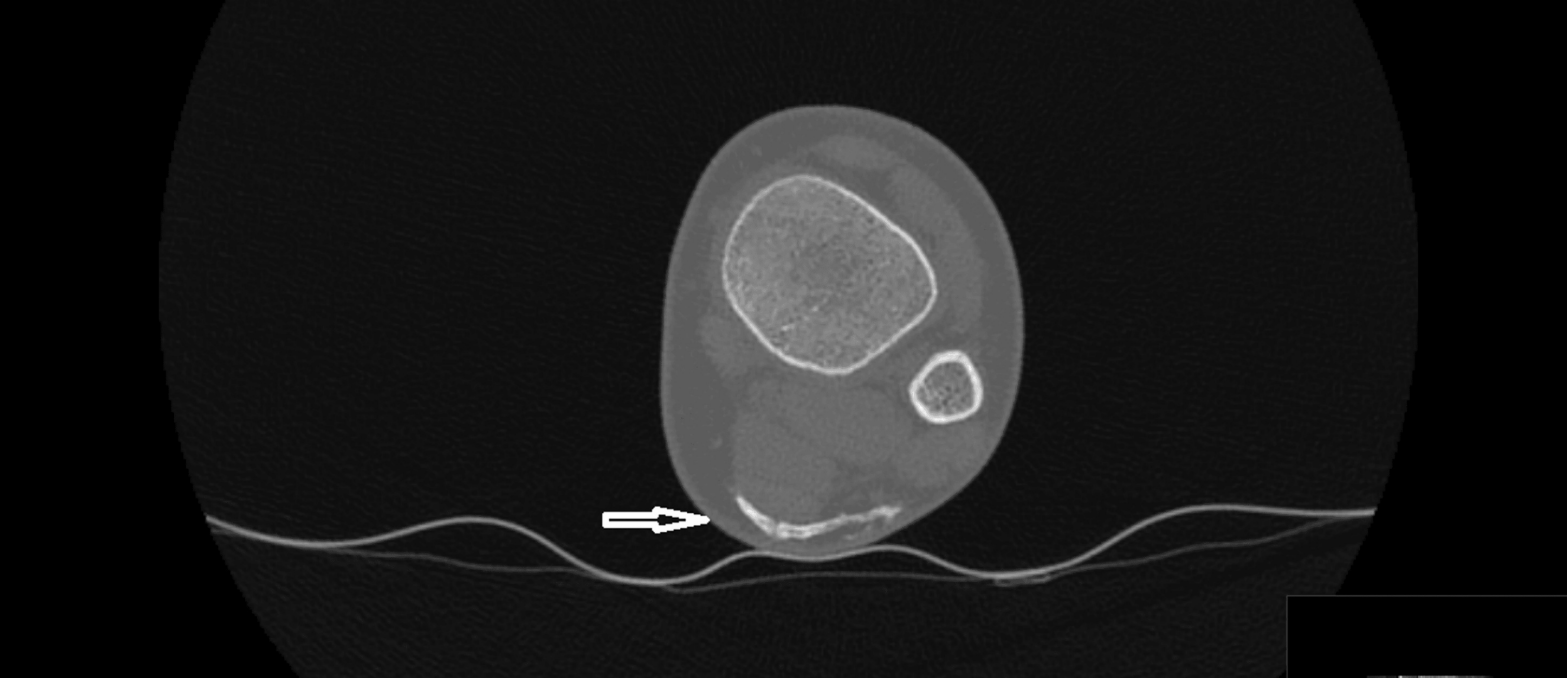
CT image on the axial plane showed a thin calcified lesion, located above the Achilles tendon, extending toward the sural nerve and vessel.

CT scans revealed a thin calcified lesion extending toward the area of the sural nerve and vessel, overlying the Achilles tendon (Figures 5, 6).

Routine blood tests, including serum calcium and phosphorus levels, returned within normal limits. Following consultation with the patient’s parents, an excisional biopsy of the lesion was performed.

**Figure 7:**
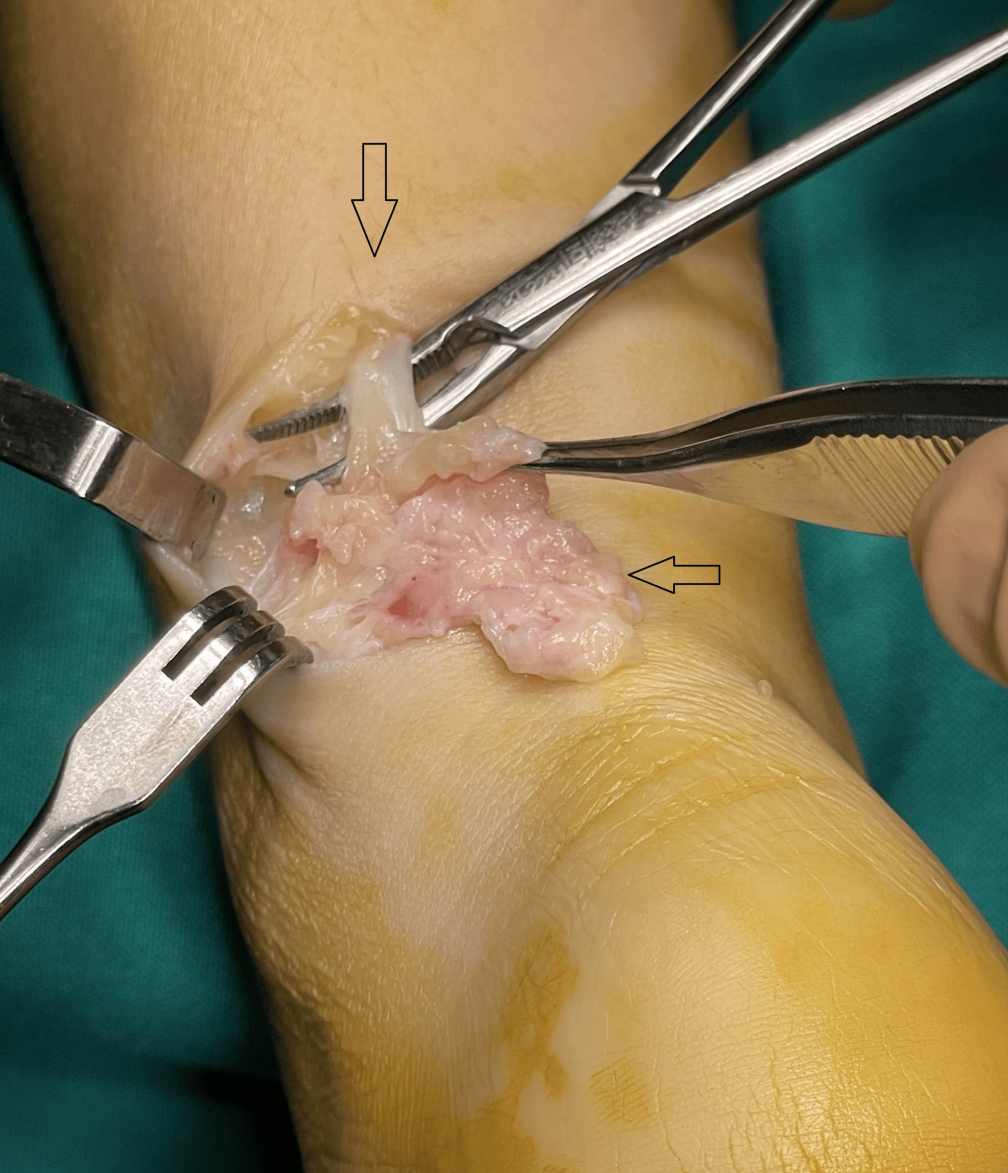
The hard mass is located just above the sural nerve and vein. Arrows are pointing the sural nerve and the mass

Under general anesthesia, with the patient in the prone position and a tourniquet applied, a longitudinal incision was made along the lateral border of the Achilles tendon. Using an operating microscope, we identified a dense, hard, irregular mass located just above the sheath of the Achilles tendon. The lesion was in direct contact with the sural nerve and accompanying vein and lacked a surrounding capsule. The entire mass was excised with care to preserve the integrity of the sural nerve (Figure 7).

**Figure 8:**
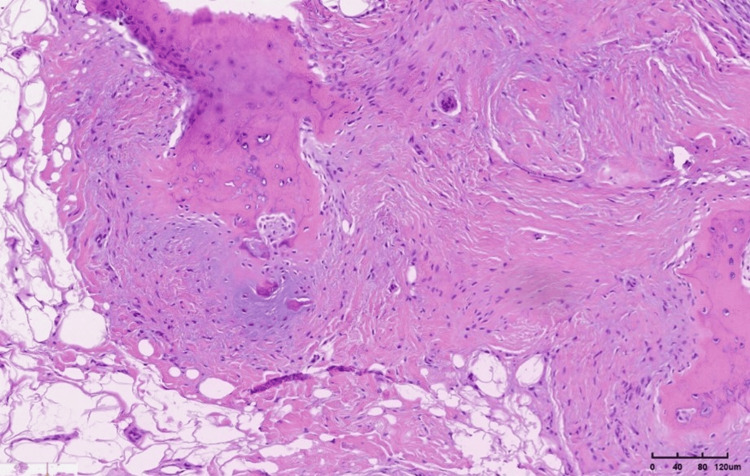
Histopathological image showing fibrous connective tissue with osteoid islands and trabeculae formation.

Histopathological examination of the excised specimen revealed a mass of fibrous connective tissue with elements of fibroblastic reactive zones, multiple areas of osteoblastic reaction, and the formation of osteoid islands and trabeculae, along with some elements of chondral tissue formation. Additionally, a few multinucleated giant cells of the osteoclastic type were identified (Figure 8).

The lesion was diagnosed as a fibro-osseous pseudotumor. The patient experienced an uneventful postoperative recovery.

## Discussion

Calcification within the subcutaneous tissue in children presents a diagnostic dilemma [9,10]. Clinically, such lesions may resemble retained foreign bodies, such as glass fragments. However, true foreign body injuries are usually associated with a clear entry point on the skin and a clear history of trauma.

Radiological imaging typically reveals diffuse irregular calcification located outside the bony margins, affecting the subcutaneous tissue. MRI demonstrates heterogeneous high and low-intensity signals, clarifying the proximity to underlying tendons, nerves, and vessels.

Calcifying aponeurotic fibromas are rare benign neoplasms described in children and adolescents, primarily affecting the hand and the foot. Hamoud et al. reported a four-year-old boy with a lesion in the same anatomical area as our patient, noting similar radiographic findings. The pathology specimen demonstrated a fibrous lesion with areas of calcification but absent osteoid formation [11]. Won et al. reported a calcifying aponeurotic fibroma in the tibialis posterior of a 74-year-old man, with pathology specimens showing focal chondroid differentiation and central calcification surrounded by spindle cell proliferation [12].

Tumoral calcinosis is a rare condition characterized by the deposition of calcium phosphate and hydroxyapatite in periarticular soft tissues, typically presenting as massive, interrupted periarticular calcified areas. Kumar et al. reported tumoral calcinosis with extensive prepatellar calcinosis and intra-articular extension in a 12-year-old boy, who was treated with open excision and biopsy. Pathology revealed several calcified masses with surrounding fibrosis [13].

Dysfunction of phosphorus regulation necessitates biochemical analysis of serum calcium, phosphorus, parathyroid hormone, and renal function tests. Literature includes reports of both hyperphosphatemia and normophosphatemic tumoral calcinosis. Perkins et al. described a case of tumoral calcinosis affecting the anterior cruciate ligament of a 13-year-old boy, initially diagnosed as chondroblastoma. However, the pathology specimen revealed an amorphous calcified material separated by bands of fibrous tissue, confirming the diagnosis of tumoral calcinosis [14].

A fibrous osseous pseudotumor is a rare benign lesion of the subcutaneous tissue, predominantly affecting the digits. Few reports exist in the literature regarding this rare entity affecting adults, with a higher prevalence in women. The clinical presentation typically involves a hard subcutaneous mass that, in case of rapid progression, may be misdiagnosed as a malignant tumor. There is no specific examination characteristic of this entity. Diffuse irregular calcification is found in the subcutaneous tissue, while a CT scan demonstrates the extent and the dimensions of the calcified tissue. MRI shows irregular areas of hyperintensity on T2-weighted images and hypointensity on T1-weighted images. Pathology examination is characterized by foci of osteoid formation surrounded by fibroblastic proliferation [15-18].

Clinical and histological features of FOP are similar to those of myositis ossificans. Myositis ossificans typically occurs in young children, primarily in deep tissues rather than in the subcutaneous tissue. The zonal phenomenon, characterized by a core of fibroblastic tissue surrounded by a layer of superficial osseous tissue, is the characteristic finding. Bizarre parosteal osteochondromatous proliferation has also been described, primarily affecting the digits of adults, and should be included in the differential diagnosis [10,19].

FOP is a rare and severe genetic disorder characterized by extensive heterotopic ossification of skeletal muscles, tendons, ligaments, and fascia. It typically presents with toe abnormalities and various osseous formations, usually starting from the dorsal axial cranial neck and extending to cover the entire body (stone man). Our patient presented with a lesion solely in the area above the Achilles tendon; there was negative familial predisposition, and her feet were normal [7,8,20].

## Conclusions

We present a unique case, to the best of our knowledge, of a fibrous osseous pseudotumor of the calf, located near the insertion of the Achilles tendon, affecting a five-year-old girl. It is crucial to perform adequate investigation for cases of subcutaneous calcification in children. Excision of the entire lesion is the recommended treatment method.
